# Antituberculosis Drug-Induced Liver Injury with Autoimmune Features: Facing Diagnostic and Treatment Challenges

**DOI:** 10.1155/2017/5741896

**Published:** 2017-01-02

**Authors:** Maria Adriana Rangel, Isabel Pinto Pais, Raquel Duarte, Isabel Carvalho

**Affiliations:** ^1^Paediatrics Department, Centro Hospitalar de Vila Nova de Gaia e Espinho, Vila Nova de Gaia, Portugal; ^2^Paediatric Gastroenterology Unit of Paediatrics Department, Centro Hospitalar de Vila Nova de Gaia e Espinho, Vila Nova de Gaia, Portugal; ^3^Pulmonology Department, Centro Hospitalar de Vila Nova de Gaia e Espinho, Vila Nova de Gaia, Portugal; ^4^The Multiresistant Tuberculosis Reference Center of the North Region of Portugal, Vila Nova de Gaia, Portugal; ^5^Department of Clinical Epidemiology, Predictive Medicine and Public Health, University of Porto Medical School, Porto, Portugal; ^6^Allergy and Pulmonology Pediatrics Unit of Pediatric Department, Centro Hospitalar de Vila Nova de Gaia e Espinho, Vila Nova de Gaia, Portugal; ^7^Pediatric Tuberculosis, Pneumologic Diagnosis Center, Vila Nova de Gaia, Portugal

## Abstract

The authors present a case report of antituberculosis drug-induced liver injury that offered diagnostic challenges (namely, the possibility of drug-induced autoimmune hepatitis) and treatment difficulties.

## 1. Introduction

Drug-induced liver injury (DILI) is a well-established concern in the treatment of tuberculosis infection and can vary widely from a transient hepatic adaptation to acute hepatitis and hepatocellular injury [1, 2]. It can seriously contribute to nonadherence, eventually contributing to treatment failure, relapse, or the emergence of drug resistance.

## 2. Case Report

Seventeen-year-old girl, Guinea native, living in Portugal, was screened for tuberculosis after pulmonary tuberculosis was diagnosed in a girl living in the same school residence. She had a positive tuberculin skin-test and interferon-gamma release assay, and a nodular image on the chest X-ray.* Mycobacterium tuberculosis* was isolated on the sputum, susceptible to all first-line antituberculosis drugs. Treatment with isoniazid, rifampicin, pyrazinamide, and ethambutol (HRZE) was started, with daily-observed administration, achieving negative sputum cultures in the first month of treatment.

Three weeks after starting treatment, elevation of Alanine Aminotransferase (ALT) and aspartate aminotransferase (AST), 136 and 89 UI/L, respectively, and palmoplantar desquamation was first detected, with further increase (ALT 281 IU/L and AST 186 IU/L) by the 5th week of treatment, leading to the interruption of the classic scheme and initiation of a second-line one with amikacin, levofloxacin, and ethambutol.

By the third month of treatment, maintaining changes on serum transaminases, despite the changes on treatment, without jaundice, cholestasis, or liver dysfunction, she was referred and admitted by the first time to our hospital. At this point, she had 31 doses of HRZE, 9 days without treatment followed by 34 doses of amikacin, levofloxacin, and ethambutol.

With a suspected DILI, withdraw of the antituberculosis drugs was determined and other causes of liver injury were excluded, such as viral hepatitis, concomitant HIV infection, alcohol consumption, and the use of other hepatotoxic offenders. During investigation, an *α*-thalassemia trait (-alpha/-alpha homozygotic), Hope Hemoglobin in beta chains, and hepatitis B immunity due to past infection (HBs and HBe antigen negative; anti-HBs and anti-HBe antibody positive) were diagnosed. Without treatment, liver enzymes rapidly normalized. Progressive introduction of antituberculosis HRZE was then made: gradual increase in the dose each day until therapeutic dose was reached; at the third day of each drug, serum ALT and AST were measured and then another drug was added. By 12th day of treatment, because of nausea complaints, worsening of the palmoplantar desquamation, and a sevenfold increase of ALT (283 IU/L), pyrazinamide was stopped. However, there was still clinical and analytical deterioration, with a peak serum ALT of 600 IU/L and AST of 300 IU/L, and all antituberculosis drugs were suspended five days later.

Further investigation to rule out autoimmune and metabolic causes was completed, all negative with exception of a positive anti-smooth-muscle antibody (ASMA 1/80). Intradermal skin tests with HRZE tested positive for rifampicin, raising the possibility of DILI by mechanisms of hypersensitivity.

Considering the patient previous treatment schemes, and based on expert's opinion, after normalization of liver enzymes, a slower reintroduction of second-line drugs was made: starting with levofloxacin 750 mg/day and amikacin 750 mg/day, twenty days later adding ethionamide 500 mg/day and 27 days later adding cycloserine 500 mg/day. There was no elevation on serum transaminases, the palmoplantar desquamation got progressively better, and she remained asymptomatic. She was discharged having 37 doses of levofloxacin plus amikacin, 17 doses of ethionamide, and 9 doses of cycloserine.

Two months later, during follow-up visits, she complained again of nausea, anorexia, weight lost, pruritus, and palmoplantar desquamation. Serum transaminases increased again (ALT 466 IU/L and AST 656 IU/L; normal alkaline phosphatase) and a mild hypergammaglobulinaemia (1580 mg/dL) with normal IgA levels (135 mg/dL), positive anti-nuclear (ANA 1/80), anti-neutrophil cytoplasmic (ANCA 1/80), smooth-muscle (ASMA 1/40), and F-actin (23.3 U) antibody were detected, raising the suspicion of drug-induced autoimmune hepatitis. Other organ autoimmune injuries (namely, thyroiditis and celiac disease) were negative. Liver biopsy showed mild lymphocytic infiltrate on histology, with no signs of interface hepatitis or portal plasma cell infiltration. Focal lymphocytic infiltrate and reticular collapse, reflecting focal hepatocytic necrosis, was evident at a lobular level.

Facing a possible immune-mediated DILI, treatment with the same antituberculosis drugs was reestablished simultaneously with prednisolone 1 mg/kg/day (40 mg/day) and maintained for a month, followed by a gradual decrease until a dose of 5 mg/day of prednisolone was reached, showing clinical improvement, no signs of liver cytolysis, normalization of hypergammaglobulinaemia, and negative autoantibodies. While still under treatment, 5 mg in alternate days was tried but liver enzymes slightly increased (ALT 42 U/L and AST 52 U/L), so she maintained 5 mg/day until 18th months of TB treatment was completed, with no side effects of steroids. After that gradually reduction of prednisolone dose to complete suspension was accomplished, maintaining normal liver enzymes and with no relapses.

## 3. Discussion

As mentioned earlier, DILI can vary from a mild transient elevation of ALT and AST, usually asymptomatic, to acute hepatitis or even liver failure [1]. The risk of hepatotoxicity ranges from 2 to as high as 33% in some studies, and it is influenced by multiple cofactors, such as drug regimen, age, alcohol consumption, malnutrition, concomitant HIV, and hepatitis B or C chronic infection [1, 2].

Towards 3 to 5 times' increase in serum transaminases, hepatotoxic drugs should be stopped, other causes ruled out, and a rechallenge made [1]. In this particular case, rechallenge was unsuccessful since symptoms rapidly relapsed (by the 12th day). Palmoplantar desquamation was interpreted as a possible immune-mediated reaction and not just a metabolic idiosyncratic reaction to drugs [4]. Investigation pointed out a possible delayed hypersensitivity reaction to rifampicin [5]. Since it was not possible to use the first-line drugs, namely, HRE, and because of the fast and severe relapse on the first rechallenge, a slower reintroduction of a complete alternative scheme was attempted. The second-line drugs are often used in special conditions like resistance to first-line therapy, extensively drug-resistant tuberculosis (XDR-TB), and have not been tested systematically. These include (1) aminoglycosides such as amikacin and kanamycin; (2) polypeptides such as capreomycin, viomycin, and enviomycin; (3) fluoroquinolones such as ciprofloxacin, levofloxacin, and moxifloxacin; (4) thioamides such as ethionamide and prothionamide; (5) cycloserine; and (6) terizidone [6]. Expert opinion suggests that a regimen of this sort should be given for at least 18–24 months [6–8].

The success of a TB treatment plan will depend on combining an appropriate number of drugs (usually at least four) and selecting one with the ability to kill* M. tuberculosis* in its various stages of growth. Among second-line drugs, only the fluoroquinolones (especially new-generation) and injectables have good bactericidal activity, followed by the thioamides. It is possible that the new fluoroquinolones will also have a sterilising capacity on these bacilli [9]. Hence, although fluoroquinolones are sometimes associated with mild, transient elevations in aminotransferase levels [10], the patient started a second-line treatment with levofloxacin plus other hepatic-safer drugs, with no elevation on serum transaminases, palmoplantar desquamation getting progressively better, and no other symptoms.

Two months later, nevertheless, she again developed symptoms associated with serum transaminases elevation, the same liver injury phenotype from previous DILI, but, at this time, with hypergammaglobulinaemia and positive autoantibodies, namely, ANA, ASMA, pANCA, and F-actin, raising the hypothesis of drug-induced autoimmune hepatitis [11, 12]. A second episode of DILI in the same patient is extremely rare (~1,2% of patients) and the probability of making a diagnosis of AIH seams to be increased in the second episode [13]. In this particular patient, 2nd DILI occurred later (more than 2 months after initiating this second-line treatment), while completely different and relatively safe-hepatic drugs were used and with autoimmune features. There are at least three clinical scenarios proposed for DILI with autoimmune features (Table 1): AIH with superimposed DILI, drug-induced AIH (DI-AIH), and immune-mediated DILI (IM-DILI) [3]. There is significant overlap of clinical and histological features between them and, in some instances, mixed features of DI-AIH and IM-DILI, as well as DILI with positive autoantibodies [3]. Frequency of DI-AIH is variable (2,15–17%) and may be explained by the fact that it is often misdiagnosed and underreported [3].

Although AIH diagnosis was not supported by the liver histology, and the scoring for AIH of the* International Autoimmune Hepatitis Group* was below 10 (probable diagnosis for scores 10–15; and definitive diagnosis for scores > 15) [11], with a low dose of prednisolone, patient improved and remained asymptomatic, proving to be beneficial and allowing tuberculosis treatment. Since patient remained asymptomatic and with normal liver enzymes afterwards, IM-DILI is the more likely diagnosis.

## Figures and Tables

**Table 1:** Classification of drug-induced autoimmune liver disease.

AIH with DILI	Patients with known AIH AIH quiescent: the drug may be the trigger of a new bout AIH under IS or corticosteroids treatment: reactivation of a known AIH upon intro of a new drug (very difficult to demonstrate a causal relationship as it might be coincidental) Often advanced fibrosis on histology

DI-AIH	Patient with a low-grad disease not diagnosed before or predisposition to AIH Drug produce an immune reaction that lead to a chronic process: perpetuating the AIH Permanent need of IS Habitually typical HLA-DR associated

IM-DILI (Autoimmune hypersensitivity)	Fever, eosinophilia, lymphadenopathy, rush Indistinguishable from true AIH: mandatory IS treatment Frequently spontaneous remission after drug cessation Usually complete response to treatment and sustained remission without relapse It is the most frequent drug-induced immune process in the liver attributable to drugs

AIH: autoimmune hepatitis; DILI: drug-induced liver injury; IS: immunosuppressants; IM-DILI: immunomediated DILI; DIAILD: drug-induced autoimmune liver disease; HLA: human leukocyte antigen.

Adapted from [3].

## Additional Points

*Summary*. This case report illustrates a hepatotoxic reaction developing during treatment of TB very difficult to manage and its diagnostic challenges. Although susceptible to all first-line antituberculosis drugs and with excellent response to treatment (negative cultures in the first month of treatment and normal chest X-ray), these side effects required the use of second-line regimens, witch are longer in duration and usually associated with the use of an injectable drug. The associated morbidity can severely compromise the adherence to treatment.
